# Association Between Serum Uric Acid and Atrial Fibrillation in Patients With Hypertension: A Systematic Review and Meta-Analysis

**DOI:** 10.31083/RCM28168

**Published:** 2025-05-27

**Authors:** Nianping Tang, Zhouling Luo, Yao Ye, Gang Wu

**Affiliations:** ^1^Department of Geriatrics, The Fourth Affiliated Hospital of Guangxi Medical University, 545005 Liuzhou, Guangxi, China

**Keywords:** uric acid, atrial fibrillation, marker

## Abstract

**Background:**

Atrial fibrillation (AF) and hypertension are associated with inflammatory response and oxidative stress. Uric acid (UA) is the product of the oxidative reaction and a surrogate indicator of oxidative stress. However, whether UA imposes a greater risk of AF in hypertensive patients remains unclear. This study sought to evaluate the evidence supporting an association between serum uric acid (SUA) and AF in patients with hypertension.

**Methods:**

The observational studies in which SUA was measured and AF was reported in hypertension were searched for in the PubMed, Cochrane Library, EMBASE, and Web of Science databases until December 31, 2023, without language restrictions. We calculated the pooled mean difference of SUA between those with and without AF in the hypertension patients.

**Results:**

A total of 5 studies were included. Three cross-sectional studies comprised 4191 patients with hypertension. The standardized mean difference (SMD) of SUA for those with AF was 0.60 (95% confidence interval (CI) 0.15–1.05) compared with those without AF. Two cohort studies evaluated 9810 individuals with hypertension, and the risk of AF was 0.03 (95% CI –0.05–0.11), which revealed no significant difference between high SUA and normal SUA.

**Conclusion:**

Our findings demonstrate a significant association between SUA and AF in patients with hypertension. Further studies are needed to investigate the underlying pathophysiological mechanisms and to assess the value of SUA as a marker or a potential target for the therapy of AF in patients with hypertension.

## 1. Introduction

Atrial fibrillation (AF) is a cardiac arrhythmia associated with an increased risk of stroke, dementia, and heart failure. AF is an important cause of mortality and morbidity [1], and considerable research effort has been focused on its pathogenesis, prevention and treatment. The development of AF is a complicated and multifactorial process. Several risk factors have been identified, including aging, male gender, congestive heart failure, kidney disease, and diabetes mellitus [2, 3]. Another major contributor to mortality and morbidity is hypertension, which often coexists with AF [4]. Epidemiological studies suggest that hypertension is also an important risk factor for AF. Moreover, accumulating evidence indicates that inflammation and oxidative stress are involved in the pathogenesis of both AF and hypertension [5, 6].

In humans, uric acid (UA) produced by xanthine oxidase (XO) is the end product of purine metabolism [7]. Several studies suggest that an elevated serum UA (SUA) level is positively associated with many common diseases, including cardiovascular diseases (CVD) [4, 8]. In particular, the association between SUA and AF is well recognized [9, 10, 11]. A number of cross-sectional, cohort, and interventional studies have also reported that elevated SUA is an independent risk factor for hypertension [12, 13, 14, 15].

Elevated SUA is associated with many common cardiovascular risk factors, such as obesity, insulin resistance and dyslipidemia. Moreover, elevated SUA is a component of metabolic syndrome, although it is unclear whether this is a cause or consequence of hypertension and AF [4, 8]. Thus, it is still uncertain whether elevated SUA poses a greater risk for AF in hypertensive patients. There has been no systematic review of the evidence in relation to this topic, the aim of the present study was therefore to systematically review the association between SUA and AF in hypertensive patients and to conduct a meta-analysis of the published evidence. The results should help to better understand the relevant factors underlying AF and improve the prevention and treatment of this condition.

## 2. Methods

### 2.1 Search Strategy

We searched the PubMed, Embase, Cochrane and Web of Science databases for relevant literature published up to December 31, 2023 using a broad search strategy that included the keywords: (“uric acid” OR “hyperuricemia” OR “urate”) AND (“atrial fibrillation” OR “atrial flutter”) AND (“hypertension” OR “high blood pressure”). There were no geographic or language limitations, and two researchers searched the databases independently. Our systematic review followed the guidelines outlined in the Preferred Reporting Items for Systematic Reviews and Meta-Analyses (PRISMA).

### 2.2 Selection Criteria

All related studies identified from the electronic databases were imported to EndNote. Two investigators (NPT, ZLL) independently reviewed and screened the titles and abstracts to eliminate irrelevant articles. The same two investigators then independently evaluated potential papers by reading the full text. Any uncertainty regarding the inclusion of an article was resolved through consensus and consultation with a third investigator (YY). Papers were included if they met the following criteria: (1) the study was aimed primarily at investigating the association between UA and AF, including participants with hypertension; (2) the sample size for the study was >100 cases, with a follow-up time of at least one year. Cohort studies, randomized controlled trials, retrospective studies and cross-sectional studies were eligible, but case reports, animal experiments, reviews and conference abstracts were excluded. Studies that lacked the necessary data for statistical analysis were also excluded. Reference lists from the retrieved full text citations were carefully screened for other eligible studies.

### 2.3 Data Extraction and Quality Assessment

One investigator (NPT) extracted the data, which included the first author, study characteristics, and essential study information. Participant characteristics and harms data from all potentially relevant studies were entered into a standardized evidence table. A second investigator (ZLL) checked the data for accuracy, and any inconsistencies were resolved by discussion with a third investigator (YY). For studies with insufficient information, the investigators contacted the primary authors wherever possible to acquire and verify the data.

Both cross-sectional studies and cohort studies were included in this meta-analysis. Two investigators (NPT, ZLL) applied the 22-item STROBE checklist to perform quality assessment [16]. A third investigator (YY) resolved any disagreements regarding the abstracted data. These items relate to the article’s title and abstract (item 1), introduction (items 2 and 3), methods (items 4–12), results (items 13–17), and discussion (items 18–21) sections, as well as other information (item 22 on funding). Eighteen items are common to the two designs, while four items (6, 12, 14, and 15) are design-specific, with different versions for all or part of the item. A score of 1 was assigned to the item if it had been met appropriately, and 0 if it had not. This score was then added to the total score [9]. The maximum possible score for cohort studies was 33, and for cross-sectional studies it was 32.

### 2.4 Statistical Analysis

Some of the studies in this meta-analysis reported SUA in mg/dL. This value was converted using a conversion rate of 16.81 (1 mg/dL = 59.48 mmol/L).

Rev-Man software (version 5.3, Cochrane Collaboration, Oxford, UK) was used to analyze and synthesize the extracted data. For all cohort and cross-sectional studies, continuous data was used to estimate the association between SUA level and AF in participants with hypertension. In studies in which AF was grouped, we combined the mean and variance of UA in each group. In studies with subgroups, we used a combination of mean and variance to combine the mean and variance of the subgroups. The standardized mean difference (SMD) was calculated, with a 95% CI in SUA between patients with or without AF in those with hypertension. The SMD is the difference between the weighted mean and SD of the SUA of individuals with AF and the controls. Cochran’s Q statistic with a *p* value < 0.10, and I^2^ statistic with a cut-off ≥50%, were used to assess statistical heterogeneity [8]. A random effects model was used when significant heterogeneity was found among the studies. Sensitivity analyses were conducted in which the SMD was recalculated by omitting one study at a time.

## 3. Results

### 3.1 Search Results

**Fig. 1.**
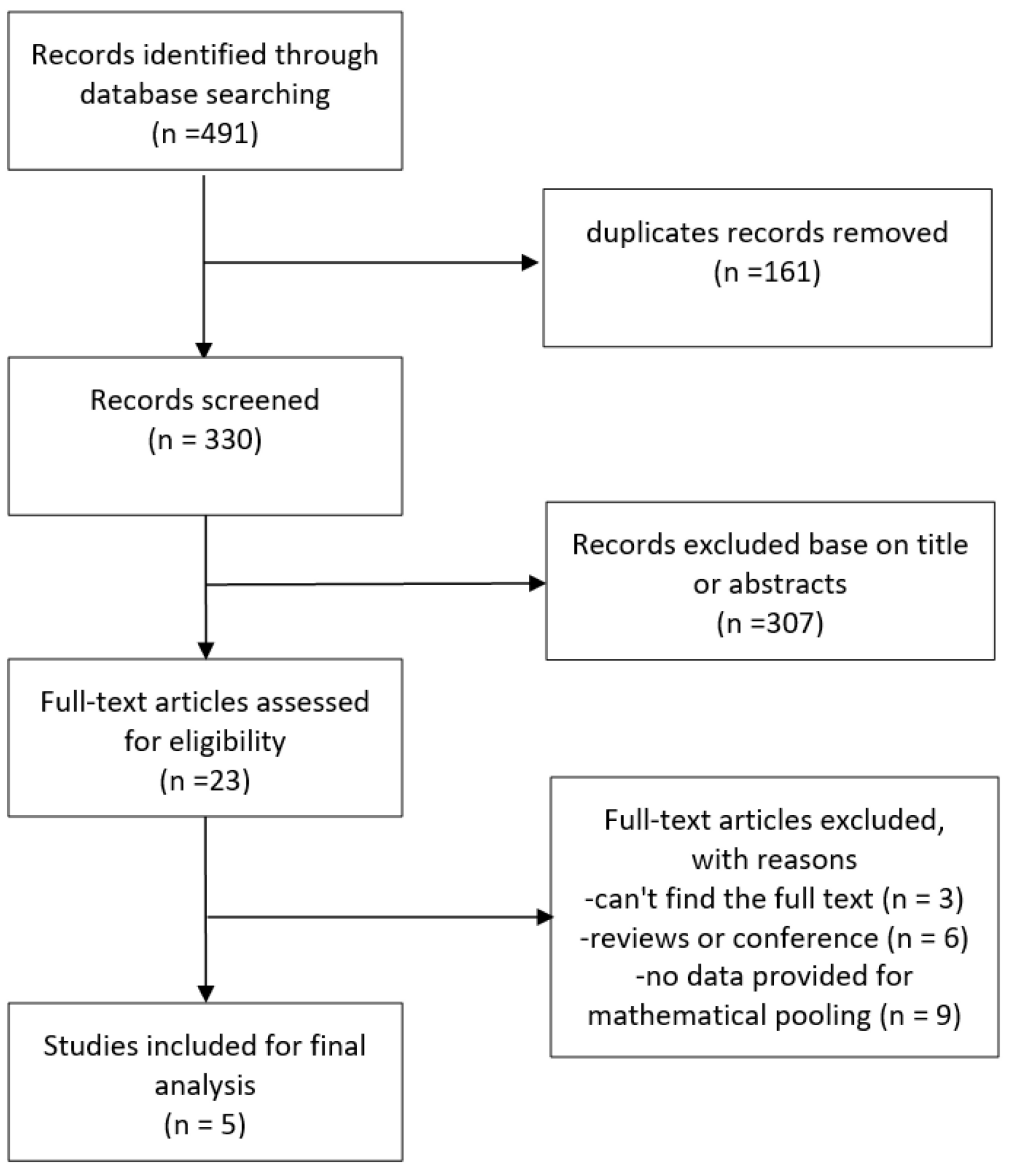
**Flowchart for the study selection**.

The study selection process is shown in Fig. 1. The search strategy identified 491 articles in total, of which 330 remained after excluding duplications. Following screening of the titles and abstracts, a further 307 were excluded, leaving 23 papers that were potentially relevant. After reviewing the full text, 5 articles met the predefined inclusion criteria, of which two were cohort studies [17, 18] and three were cross-sectional studies [19, 20, 21].

### 3.2 Characteristics of the Studies

Table 1 (Ref. [17, 18, 19, 20, 21]) presents the baseline characteristics of the 5 studies, all of which were retrospective analyses. A total of 14,001 high blood pressure (HBP) patients were analyzed, with a sample size that ranged between 268 and 8831 for each study. The mean age of patients ranged from 55.7 to 71.8 years. In the methodological quality assessment, scores for the 22-item STROBE checklist were 22 or 23 points for the three cross-sectional studies, and 23 and 25 for the two cohort studies [17, 18, 19, 20, 21].

**Table 1.** **Characteristics of the five included studies**.

First author (year)		Sample size (% of men)	Median follow-up year (years)	Age (years)	No. of patients with AF	Mean SUA of patients with AF (µmol/L)	No. of controls	Mean SUA of controls (µmol/L)	Quality score of study
Cohort									
	Chuang (2014) [17]	979 (—)	9.16	71.8 ± 5.3	52	424.6 ± 125.6	18	394.7 ± 107.6	23
	Okin (2015) [18]	8831 (—)	4.6	67 ± 7	701	331 ± 75	8130	329 ± 78	25
Cross-sectional									
	Hu (2010) [19]	3472 (50.8)	—	67	125	418.6 ± 101.7	3347	382.7 ± 143.5	23
	Liu (2011) [20]	451 (49.4)	—	55.7 ± 11.8	50	368.9 ± 100.7	401	314.6 ± 92.9	22
	Shi (2016) [21]	268 (49.6)	—	71.0 ± 8.8	132	379.1 ± 122.3	136	281.5 ± 70.5	22
Total		14,001			1060		12,032		

SUA, serum uric acid; AF, atrial fibrillation.

### 3.3 Relationship Between AF and SUA

**Fig. 2.**
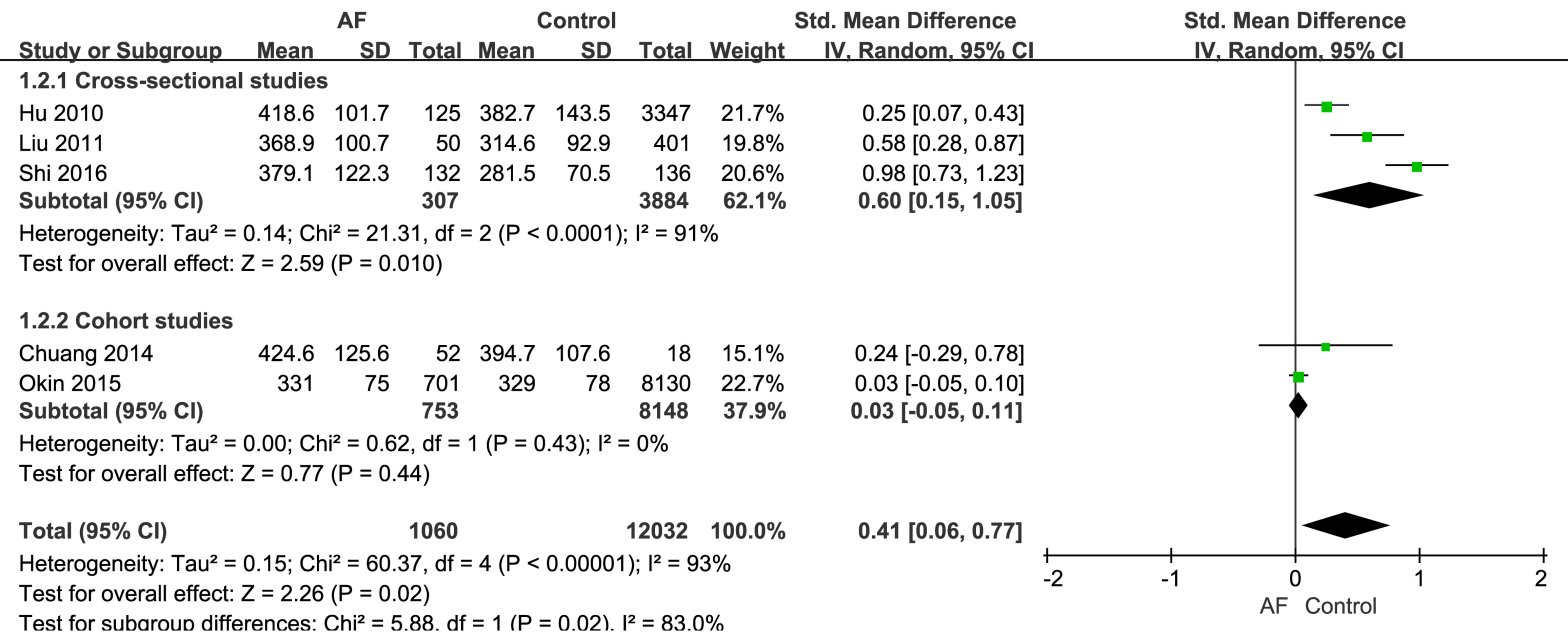
**The relationship between AF and SUA**. SD, standard deviation; IV, inverse variance.

The three cross-sectional studies found that SUA was a predictor of AF [19, 20, 21], with SUA levels being significantly higher in the AF group than the control group (Fig. 2). Moreover, the SMD of SUA in AF patients was 0.60 (95% CI: 0.15–1.05), which was significantly different to the control group (*p* = 0.01) in the mathematical pooling. However, significant heterogeneity (I^2^ = 91%, *p* < 0.01) was observed, mainly because one of the studies had an SMD of 0.98.

The mathematical pooling for the two cohort studies is also shown in Fig. 2. The SMD of SUA in the AF group was 0.03 (95% CI: –0.05–0.11), with no significant degree of heterogeneity and no significant difference between the AF and control groups (*p* = 0.44).

Our meta-analysis indicated that SUA is associated with AF in individuals with hypertension. Elevated UA levels significantly increased the risk of AF by a SMD of 0.41 (95% CI: 0.06–0.77, *p* = 0.02). Statistical heterogeneity (I^2^ = 93%, *p* < 0.01) was observed, and a randomization model was constructed.

### 3.4 Subgroup Analysis

The sources of heterogeneity within subgroups could not be evaluated due to the small number of events in some of the studies.

### 3.5 Publication Bias

Funnel plot analysis to examine publication bias could not be performed due to the small number of included studies.

## 4. Discussion

In the present study, we showed that elevated SUA is associated with an increased risk of AF in hypertensive patients. Intriguingly, although the result supports our hypothesis, this association was only seen in the three cross-sectional studies [19, 20, 21] and not in the two cohort studies [17, 18]. AF is a multifactorial disease and its development is a complicated process affected by many risk factors, including SUA level and hypertension. Moreover, the risk factors may also interact with each other. Cross-sectional studies only provide clues regarding the cause of disease, and the associations may not be as strong as in cohort studies with a temporal causal order. Thus, the inconsistency between the two types of study could be due to the influence of temporal factors in the cohort studies.

Several limitations of this study should be mentioned. First, substantial heterogeneity was observed in the relationship between SUA and AF in the five studies [17, 18, 19, 20, 21] included in this analysis. However, the source of this heterogeneity could not be identified and it may have skewed the results. Secondly, the number of analyzed studies was small and none were prospective. Hence, the inherent limitations of the original studies cannot be avoided and we can only speculate on the association between SUA and AF. Thirdly, patients enrolled in the evaluated studies were from an elderly population, and we do not know whether a similar conclusion also applies to younger patients. However, our results were obtained following a stringent analysis, and represent the first systematic review and meta-analysis of published studies to estimate the association between SUA and AF in hypertensive populations. These findings provide valuable insights for the treatment and prevention of AF in hypertensive patients.

Epidemiological studies suggest that elevated SUA is related to the occurrence and development of AF [10, 22, 23]. Moreover, previous studies [10, 22, 23] have shown correlations between SUA and AF both in the presence and absence of diabetes mellitus and other complications. In other words, SUA is considered to be an independent risk factor for AF [24, 25]. Siliste *et al*. [26] found that SUA was associated with AF in women with metabolic syndrome, but not in men with this condition. These authors also reported that SUA was an independent predictor of AF.

Nevertheless, AF has a complex origin and multiple pathways of initiation. The definitive mechanisms underlying the development of AF remain to be elucidated, although independent risk factors have been identified and its pathophysiology has been extensively studied.

Left atrial (LA) enlargement is one of the pathological manifestations of AF, as well as being an independent risk factor for this condition. LA enlargement with a consequent decrease in LA function indicates maladaptive structural and functional remodeling. This in turn leads to electrical and ionic remodeling. Atrial remodeling is essential for arrhythmia initiation and perpetuation in the majority of AF cases, and is therefore considered to be a plausible explanation for the occurrence of AF [27]. Results from experimental studies show that oxidative stress and inflammatory mediators such as UA can induce electrophysiological and structural remodeling of atrial and ventricular myocardium. This is achieved through fibroblast proliferation, inflammation and apoptosis, as well as intracellular calcium overload and a reduction in sodium channels [28, 29]. Furthermore, a recent study found a correlation between UA and pathological substrates in the left atrium, providing an additional perspective into the mechanism of AF [30].

UA is the end product of purine degradation in humans. It is produced by XO and is an alternative marker for oxidative stress. The accumulation of UA inside atrial cardiomyocytes can induce remodeling by elevating the levels of reactive oxygen species (ROS). XO is a key enzyme of purine catabolism and is responsible for the generation of free radicals during purine metabolism. Interestingly, previous studies have reported that allopurinol reduces oxidative stress by inhibiting XO, with the subsequent UA production being beneficial for both ventricular and atrial remodeling [31, 32, 33].

Maharani *et al*. [34] showed that soluble UA enters HL-1 atrial myocytes via a UA transporter. Intracellular UA then induces oxidative stress via activation of NADPH oxidase and subsequent activation of the MAPK/ERK pathway. This results in increased expression of the Kv1.5 channel protein through slowing of its protein degradation without altering the mRNA level. Activation of the potassium voltage-gated Kv1.5 channel shortens the duration of atrial action potential (APD), shortens the effective refractory period (ERP), leading finally to electrical remodeling and an increased risk of AF [10, 35].

Notwithstanding the correlation between high SUA and AF, multiple confounding factors other than SUA have been implicated in AF. Hypertension is one of the prevalent, independent, and potentially modifiable risk factors for AF. Chronic hypertension causes left ventricular hypertrophy and impairment of diastolic function, thereby increasing the left ventricular end-diastolic pressure. This increases the pressure and volume of the LA, leading to AF [36]. Hu *et al*. [19] reported that metabolic syndrome was not associated with a higher risk of AF in patients with hypertension, regardless of the presence or absence of left ventricular hypertrophy. Their results were similar to those of the Systolic Hypertension in the Elderly Program database (SHEP) study [37]. Similarly, the Framingham Heart Study showed that as the treatment of hypertension becomes more frequent, the incidence of severe hypertension becomes less common. However, individuals with hypertension complicated by AF are not affected by this, explaining why antihypertensive therapy does not completely eliminate the increased risk of AF associated with hypertension [38].

Most previous studies have focused only on healthy populations. The current work investigated the association between SUA and AF in patients with hypertension. Although both high SUA and hypertension proved to be independent risk factors for AF, SUA has been associated with hypertension in previous studies [39, 40, 41]. Increasing the SUA levels using uricase inhibitors led to systemic hypertension in a rat model [42, 43]. Kuwabara *et al*. [44] found that after adjusting for age, body mass index (BMI), dyslipidemia, diabetes, smoking and estimated glomerular filtration rate, the prevalence of hypertension increased 1.2-fold for every 1 mg/dL increase in the SUA level. In a subsequent retrospective cohort study [45], these authors also found the cumulative incidence of hypertension was higher in subjects with hyperuricemia than in those without. This held for both normotensive (5.6% versus 2.6%, respectively) and prehypertensive subjects (30.7% versus 24.0%, respectively). The difference in incidence of hypertension from prehypertension between individuals with hyperuricemia or normouricemia was greater in women (38.4% versus 22.8%) than in men (28.7% versus 24.5%).

The antihypertensive effect of allopurinol following its reduction of UA was investigated in a meta-analysis [46]. A subsequent meta-analysis also demonstrated that both inhibitors of UA production (allopurinol) and enhancers of UA excretion (probenecid) can lower blood pressure [47]. The positive association between SUA and hypertension makes it difficult to investigate the actual role or contribution of UA to the development of AF when complicated with hypertension. This may be the major reason behind the inconsistent results between the cross-sectional and cohort studies observed in the present analysis.

Another factor that should be considered is the type of antihypertensive drug used by the hypertensive patients. Thiazide-type diuretics, angiotensin-converting enzyme (ACE) inhibitors, and non-losartan angiotensin-II receptor blockers (ARB) can reduce renal excretion of urate, thereby increasing SUA levels [48, 49]. Beta-blockers can also increase the levels of SUA [50]. In contrast, the angiotensin-II receptor antagonist losartan and long-acting calcium channel blockers (CCB) can decrease SUA levels in patients with coronary artery disease or hypertension, resulting in protection from cardiovascular events and gout [51, 52]. In the two cohort studies included in our analysis, the effect of antihypertensive drugs was not considered, which may explain the non-significant result.

Taking this into account, it is clear that additional well-designed and large-scale studies are needed to investigate the role or contribution of high SUA to the development of AF in patients with hypertension.

## 5. Conclusion

Despite the limitations of this study, our analysis indicates a clear and strong association between AF and elevated SUA in hypertensive patients. This association was primarily observed in cross-sectional studies. Further large-scale, high-quality randomized controlled trials in different populations are needed to confirm whether reducing the SUA level can decrease the risk of AF in patients with very high blood pressure. Further studies should also focus on identifying mechanistic links between SUA and AF in hypertension so that UA-lowering agents can be used on a rational basis.

## Availability of Data and Materials

All data generated or analyzed during this study are included in this article. Further inquiries can be directed to the corresponding author.

## Supplementary Material

Supplementary material associated with this article can be found, in the online version, at https://doi.org/10.31083/RCM28168.
